# Development of a Mouse-Adapted Reporter SARS-CoV-2 as a Tool for Two-Photon In Vivo Imaging

**DOI:** 10.3390/v16040537

**Published:** 2024-03-29

**Authors:** Hiroshi Ueki, Maki Kiso, Yuri Furusawa, Shun Iida, Seiya Yamayoshi, Noriko Nakajima, Masaki Imai, Tadaki Suzuki, Yoshihiro Kawaoka

**Affiliations:** 1 Division of Virology, Institute of Medical Science, University of Tokyo, Tokyo 108-8639, Japan; h-ueki@ims.u-tokyo.ac.jp (H.U.); kisomaki@g.ecc.u-tokyo.ac.jp (M.K.); yuri-furusawa@g.ecc.u-tokyo.ac.jp (Y.F.); yamayo@ims.u-tokyo.ac.jp (S.Y.); mimai@ims.u-tokyo.ac.jp (M.I.); 2 Center for Global Viral Diseases, National Center for Global Health and Medicine Research Institute, Tokyo 162-8655, Japan; 3 Pandemic Preparedness, Infection and Advanced Research Center (UTOPIA), University of Tokyo, Minato-ku, Tokyo 108-8639, Japan; siida@niid.go.jp (S.I.); tenkonakajima@gmail.com (N.N.); tksuzuki@niid.go.jp (T.S.); 4 Department of Pathology, National Institute of Infectious Diseases, Tokyo 162-8640, Japan; 5 Department of Pathobiological Sciences, School of Veterinary Medicine, University of Wisconsin-Madison, Madison, WI 53711, USA

**Keywords:** SARS-CoV-2, COVID-19, mouse model, BALB/c, C57BL/6J, in vivo imaging, two-photon excitation microscopy

## Abstract

Severe acute respiratory syndrome coronavirus 2 (SARS-CoV-2) often causes severe viral pneumonia. Although many studies using mouse models have examined the pathogenicity of SARS-CoV-2, COVID-19 pathogenesis remains poorly understood. In vivo imaging analysis using two-photon excitation microscopy (TPEM) is useful for elucidating the pathology of COVID-19, providing pathological insights that are not available from conventional histological analysis. However, there is no reporter SARS-CoV-2 that demonstrates pathogenicity in C57BL/6 mice and emits sufficient light intensity for two-photon in vivo imaging. Here, we generated a mouse-adapted strain of SARS-CoV-2 (named MASCV2-p25) and demonstrated its efficient replication in the lungs of C57BL/6 mice, causing fatal pneumonia. Histopathologic analysis revealed the severe inflammation and infiltration of immune cells in the lungs of MASCV2-p25-infected C57BL/6 mice, not unlike that observed in COVID-19 patients with severe pneumonia. Subsequently, we generated a mouse-adapted reporter SARS-CoV-2 (named MASCV-Venus-p9) by inserting the fluorescent protein-encoding gene Venus into MASCV2-p25 and sequential lung-to-lung passages in C57BL/6 mice. C57BL/6 mice infected with MASCV2-Venus-p9 exhibited severe pneumonia. In addition, the TPEM of the lungs of the infected C57BL/6J mice showed that the infected cells emitted sufficient levels of fluorescence for easy observation. These findings suggest that MASCV2-Venus-p9 will be useful for two-photon in vivo imaging studies of the pathogenesis of severe COVID-19 pneumonia.

## 1. Introduction

Most cases of coronavirus disease 2019 (COVID-19), which is caused by severe acute respiratory syndrome coronavirus 2 (SARS-CoV-2), present with mild respiratory symptoms. However, the elderly and those with underlying medical conditions often develop severe viral pneumonia and acute respiratory distress syndrome, which can lead to death [1,2]. A large number of studies, including single-cell RNA-seq and proteomic approaches using clinical samples and animal models of SARS-CoV-2 infection, have suggested that immune responses may play an important role in the pathogenesis of severe COVID-19; however, the mechanisms by which SARS-CoV-2 causes severe viral pneumonia and occasional death are not yet fully understood [3,4,5,6].

To fully understand the pathogenesis of COVID-19 pneumonia, we need to observe the lungs of SARS-CoV-2-infected animals under physiological conditions from initial infection to the development of severe disease. Although non-invasive imaging techniques such as computed tomography [7] and IVIS Spectrum (an in vivo imaging system) [8] have been used in SARS-CoV-2 research, they are limited by their low spatio-temporal resolution and can only provide estimates of inflammatory sites within organs. Consequently, with these methods, we are unable to directly observe the cellular responses of the immune system. In contrast, the use of laser-based in vivo two-photon imaging techniques (e.g., in vivo two-photon imaging) allows the observation of cell and blood flow dynamics within organs and tissues of living animals [9,10,11]. This approach has the unique advantage of providing valuable information about cell behavior, morphology, changes in tissue localization, and hemodynamics that cannot be obtained by conventional histological analysis. To conduct in vivo two-photon imaging analyses using SARS-CoV-2-infected animals, we need a reporter SARS-CoV-2 that is pathogenic in small animal models of COVID-19 and can be visualized in infected cells.

Small animal models that mirror the pathogenesis of severe COVID-19 are needed for studies on the pathophysiologic mechanisms of severe COVID-19 and to help establish effective therapeutic measures. SARS-CoV-2 initiates infection via the interaction between its spike (S) protein and the host cell surface receptor [i.e., human angiotensin-converting enzyme 2 (hACE2)] [12,13]. Mice are a useful small animal model for infectious disease research [14]; however, the ancestral strains of SARS-CoV-2 cannot infect standard laboratory mice due to the inefficient interaction between the S proteins and mouse ACE2 (mACE2) [15]. Transgenic mice expressing human ACE2, regulated by the human cytokeratin 18 promoter in the epithelial cells, have been widely used to study SARS-CoV-2 pathogenesis [16,17]. But, the intranasal inoculation of these mice with SARS-CoV-2 causes severe neurological disease, including encephalitis, with features that differ from severe COVID-19 cases [18,19]. Syrian hamsters are highly susceptible to SARS-CoV-2 infection [20,21,22]; however, genetically engineered hamster models of human diseases and immunological tools are currently limited [23]. Since the C57BL/6 mouse is widely used as a genetic background for genetically modified mice in studies of various human diseases [24], a C57BL/6J model that develops severe pneumonia similar to that seen in COVID-19 patients is highly desirable. Several mouse-adapted (MA) strains of SARS-CoV-2 developed through serial passaging in mice and/or reverse genetics have been shown to cause severe pneumonia in C57BL/6J mice [25,26,27]; however, mouse-adapted reporter SARS-CoV-2 strains that are pathogenic to C57BL/6J mice and express fluorescent proteins in infected cells have not been established.

Here, we generated an MA strain of SARS-CoV-2 by performing sequential lung-to-lung passages in BALB/c mice, followed by in C57BL/6J mice, and established a lethal pneumonia model in C57BL/6J mice. Subsequently, we generated a mouse-adapted reporter SARS-CoV-2 by inserting the fluorescent protein-encoding gene Venus into the MA strain of SARS-CoV-2 strain and sequential lung-to-lung passages in C57BL/6 mice.

## 2. Materials and Methods

### 2.1. Cells

VeroE6/TMPRSS2 (JCRB 1819) cells [28] were propagated in 1 mg/mL geneticin (G418; Invivogen, San Diego, CA, USA) and 5 μg/mL plasmocin prophylactic (Invivogen, San Diego, CA, USA) in Dulbecco’s modified Eagle’s medium (DMEM; Thermo Fisher Scientific, Waltham, MA, USA) containing 10% Fetal Calf Serum (FCS; Thermo Fisher Scientific, Waltham, MA, USA). The human embryonic kidney (HEK) 293T cell line, which is a derivative of the 293 cell line into which the gene for the simian virus 40 T antigen has been inserted [29], was obtained from Dr. Tadashi Matsuda (Hokkaido University). HEK293T cells were cultured in DMEM supplemented with 10% FCS and maintained at 37  °C with 5% CO_2_. The cells were regularly tested for mycoplasma contamination using PCR and confirmed to be mycoplasma-free.

### 2.2. Viruses

hCoV-19/Japan/TY7-501/2021 (TY7-501) [30] was propagated in VeroE6/TMPRSS2 cells in VP-SFM (Thermo Fisher Scientific, Waltham, MA, USA). All experiments with SARS-CoV-2 were performed in enhanced biosafety level-3 containment laboratories at the University of Tokyo and the National Institute of Infectious Diseases, Japan, which are approved for such use by the Ministry of Agriculture, Forestry, and Fisheries, Japan.

### 2.3. Mouse Adaptation of SARS-CoV-2

We used 8–10-week-old female BALB/c mice (Japan SLC Inc., Hamamatsu, Japan) and 8–10-week-old female C57BL/6J mice (Japan SLC Inc., Hamamatsu, Japan) in this study. A BALB/c-adapted strain (called MASCV2-p9) was derived from a series of sequential lung-to-lung passages in BALB/c mice [31]. Briefly, BALB/c mice were intranasally infected with 10^5^ plaque-forming units (PFU) of TY7-501 under isoflurane anesthesia. On days 3–5 post-infection, the mice were euthanized and their lungs were collected and homogenized in a 2-fold volume of DMEM containing 5% FCS. The lung homogenate was clarified by centrifugation at 5000 rpm for 5 min, and the supernatant (50 μL) was then intranasally inoculated into naïve mice; this process was repeated nine times using one mouse at each passage. A C57BL/6J-adapted strain (called MASCV2-p25) was derived by sequential lung-to-lung passages in BALB/c mice followed by sequential lung-to-lung passages in C57BL/6J mice. The supernatant of the lung homogenates after 10 passages in BALB/c mice was used to perform 15 additional lung-to-lung passages in C57BL/6J mice using one mouse at each passage. A mouse-adapted reporter SARS-CoV-2 strain (called MASCV2-Venus-p9) was generated by inserting the fluorescent protein-encoding gene Venus into MASCV2-p25, followed by nine lung-to-lung passages in C57BL/6J mice using one mouse at each passage. After these passages, the virus in the supernatant of the lung homogenate was plaque-purified and amplified on VeroE6/TMPRSS2 cells to prepare a working stock. The sequence data that support this study are available in the GISAID database (MASCV2-p9:EPI_ISL_18961019, MASCV2-p25:EPI_ISL_18961018, MASCV2-Venus:EPI_ISL_18961021, MASCV2-Venus-p9:EPI_ISL_18961020). All experiments with mice were performed in accordance with the Science Council of Japan’s Guidelines for Proper Conduct of Animal Experiments. The protocols were approved by the Animal Experiment Committee of the Institute of Medical Science, the University of Tokyo (approval number PA19-72).

### 2.4. Generation of Mouse-Adapted Reporter SARS-CoV-2

The full-genome nucleotide sequence of MASCV2-p25 was assembled into the pBeloBAC11 vector to generate infectious cDNA clones under the control of a cytomegalovirus (CMV) promoter by using Gibson Assembly Master Mix (NEW ENGLAND BioLabs, Ipwich, MA, USA), as described previously [32]. The sequence encoding the fusion construct Venus-porcine tescherovirus (PTV-1) 2A proteolytic cleavage site was placed upstream of the N gene [33]. The constructed BAC was introduced into DH10B *E. coli* (Invitorogen, Waltham, MA, USA) by use of electroporation. The *E. coli* were amplified at 37 °C and the BACs were extracted using NucleoBond Xtra Maxi (TaKaRa, Kusatsu, Japan).

To recover recombinant SARS-CoV-2, the BAC encoding the full-length SARS-CoV-2 genome was transfected into HEK293T cells using TransIT-293 (TaKaRa, Kusatsu, Japan), according to the manufacturer’s protocol. At 3 days post-transfection, the supernatant containing the virus was collected and inoculated onto VeroE6/TMPRSS2 at 37 °C to prepare the virus stock. The virus titer of the stock was determined using plaque assays in VeroE6/TMPRSS2.

### 2.5. Experimental Infection of Mice

Young (6–8-week-old) and adult (18–24-week-old) female BALB/c mice (Japan SLC Inc., Hamamatsu, Japan) and C57BL/6J mice (Japan SLC Inc., Hamamatsu, Japan) were used in this study. Baseline body weights were measured before infection. Mice were intranasally inoculated with 10^1−5^ PFU (in 50 μL) of the parental virus (TY7-501), MASCV2-p9, MASCV2-p25, or MASCV2-Venus-p9. Body weight was monitored daily for 10–14 days.

### 2.6. Pathologic Examination

For pathologic examination, three mice per group were intranasally infected with 10^3^ PFU of virus. Then, 2- and 4–5-days post-infection, the nasal turbinate, lung, brain, heart, liver, kidney, intestine, and spleen were collected. Excised lung tissues were fixed in 4% paraformaldehyde phosphate buffer solution and processed for paraffin embedding. The paraffin blocks were cut into 3 µm-thick sections and then mounted on silane-coated glass slides. One section from each tissue sample was stained using a standard hematoxylin and eosin procedure; another was processed for immunohistochemical staining with a rabbit polyclonal antibody for SARS-CoV nucleocapsid protein (ProSpec; ANT-180, 1:500 dilution, Rehovot) that cross-reacts with SARS-CoV-2 nucleocapsid protein. Specific antigen–antibody reactions were visualized by means of 3,3′-diaminobenzidine tetrahydrochloride staining using the Dako Envision system (Dako Cytomation; K4001, 1:1 dilution, Glostrup). Histopathological scores of inflammation in the alveolar regions were determined based on the percentage of alveolar inflammation in a given area of a pulmonary section (area score), inflammatory cell density (density score), and the existence of pulmonary edema and/or alveolar hemorrhage (severity score) using the following scoring system: Area score 0, no inflammation; 1, affected area (≤50%); and 2, affected area (>50%). Density score 0, inflammation observed only in the high-power field; 1, inflammation observed in low-power fields; and 2, alveoli filled with inflammatory cells. Severity score 0, absence of pulmonary edema and/or alveolar hemorrhage; and 1, presence of pulmonary edema and/or alveolar hemorrhage. The total score for the five lobes was calculated for each animal. Therefore, the histopathological scores of inflammation in the alveoli for individual animals ranged from 0 to 25.

### 2.7. In Vivo Imaging of Mouse Lung

For the in vivo imaging analysis, C57BL/6J mice were intranasally infected with 10^5^ PFU of MASCV2-Venus-p9 in 100 µL of PBS. On days 2 and 5 post-infection, the in vivo imaging was performed using an LSM 980 NLO (Carl Zeiss, Jena, Germany) equipped with an infrared laser (Chameleon Vision II; Coherent), as described previously [11,34]. In brief, the infected mice were intubated under anesthesia and ventilated at a respiratory rate of 120 breaths per minute. The mice were then placed in the right lateral decubitus position, and the left lung lobe was exposed. Subsequently, a custom-made thoracic suction window was inserted between the ribs and applied to gently immobilize the lung [11,34]. Texas red dextran (Invitrogen) was injected i.v. before imaging to visualize the lung structure. To acquire images in spectral imaging mode, lasers at a wavelength of 910 nm were used for simultaneous excitation of Venus and Texas red dextran. All emitted light between 490 and 695 nm wavelengths was detected using a 20× water-immersion lens (Carl Zeiss, Jena, Germany). Spectral separation of the acquired lambda stacks was achieved using the linear unmixing function of the LSM software ZEN blue (Carl Zeiss, Jena, Germany).

### 2.8. Virus Titration

For virologic examination, four mice per group were intranasally infected with 10^3^ PFU (in 50 μL) of virus. Then, 2- and 4–5-days post-infection, the animals were euthanized and their organs (nasal turbinate, lungs, brain, heart, liver, and spleen) were collected. The virus titers in the organs were determined by use of plaque assays on VeroE6/TMPRSS2 cells. Confluent VeroE6/TMPRSS2 cells in 12-well plates were infected with 100 μL of diluted supernatant (from undiluted to 10^−5^ dilution) from the organ homogenate. The virus inoculum was removed after a 1 h incubation at 37 °C, and then a 1% agarose solution in DMEM was overlaid on the cells. After incubation for 48 h, the agar-covered cells were fixed with 10% neutral buffered formalin. The plaques were counted after removal of the agar.

## 3. Results

### 3.1. Characterization of a Mouse-Adapted Strain of SARS-CoV-2 in BALB/c Mice

**Figure 1:**
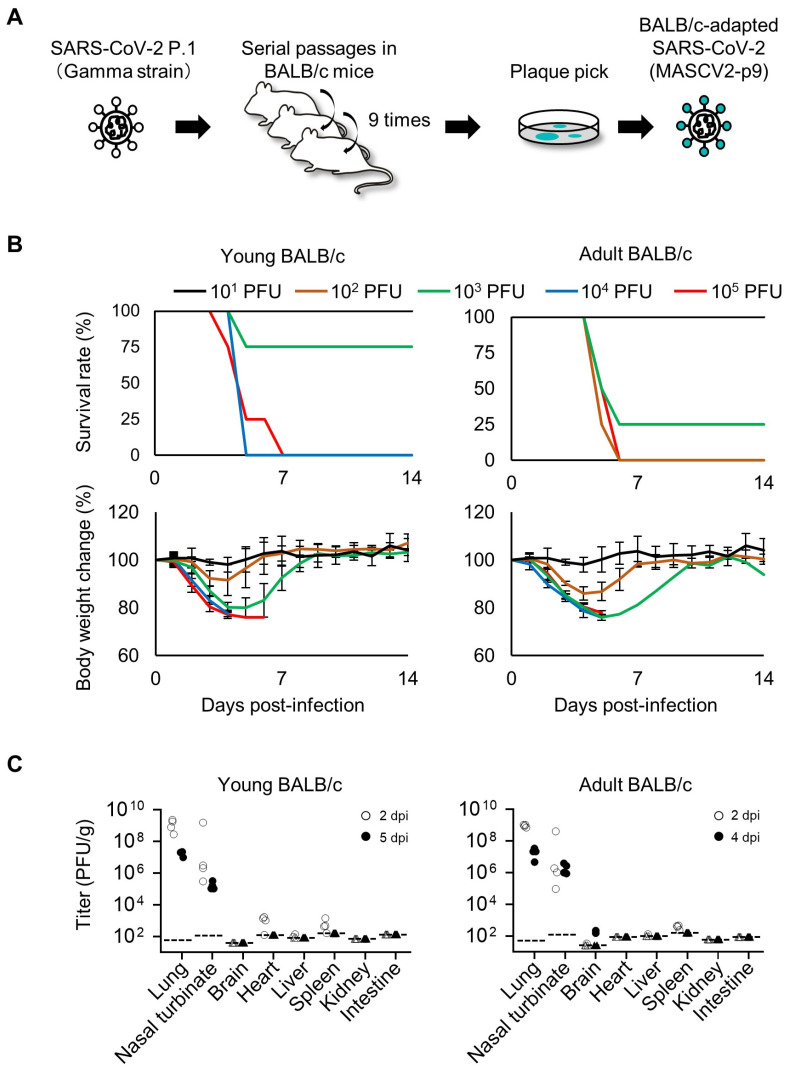
Virulence of MASCV2-p9 in BALB/c mice. (**A**) Schematic image of SARS-CoV-2 adaptation to BALB/c mice. (**B**) Survival rate and body weight changes of MASCV2-p9-infected mice. Four mice per group of young (6-week-old) or adult (18-week-old) BALB/c mice were infected with 10^1^ to 10^5^ PFU of MASCV2-p9, and survival and body weight were monitored daily for 14 days. The results are expressed as the mean ± SD. (**C**) Virus titers in organs of MASCV2-p9-infected mice. Four mice per group were euthanized and virus titers in the lungs, nasal turbinate, brain, heart, liver, spleen, kidney, and intestines were determined using plaque assays in VeroE6/TMPRSS2 cells. Dashed lines in the panels indicate the detection limit of the assay for each organ. Triangles in the panels indicate that the value was below the detection limit.

The first step in generating mouse-adapted strains of SARS-CoV-2 was to adapt the virus to BALB/c mice because of their higher susceptibility to SARS-CoV-2 compared to C57BL/6J mice [25,35,36]. To generate BALB/c-adapted SARS-CoV-2, serial lung-to-lung passages were performed, as detailed in the Methods section (Figure 1A). After the serial lung-to-lung passages, a virus in the lung homogenates was plaque-purified and amplified on VeroE6/TMPRSS2 cells to prepare a working stock of MASCV2-p9 (Table 1). Sequencing the analysis of the stock revealed that MASCV2-p9 contains an A307V substitution in NSP4, an F294L substitution in NSP5, a Q493R substitution in the S protein, and a T14I substitution in ORF7a (Table 2) compared to the initial virus stock used for infection.

**Figure 2:**
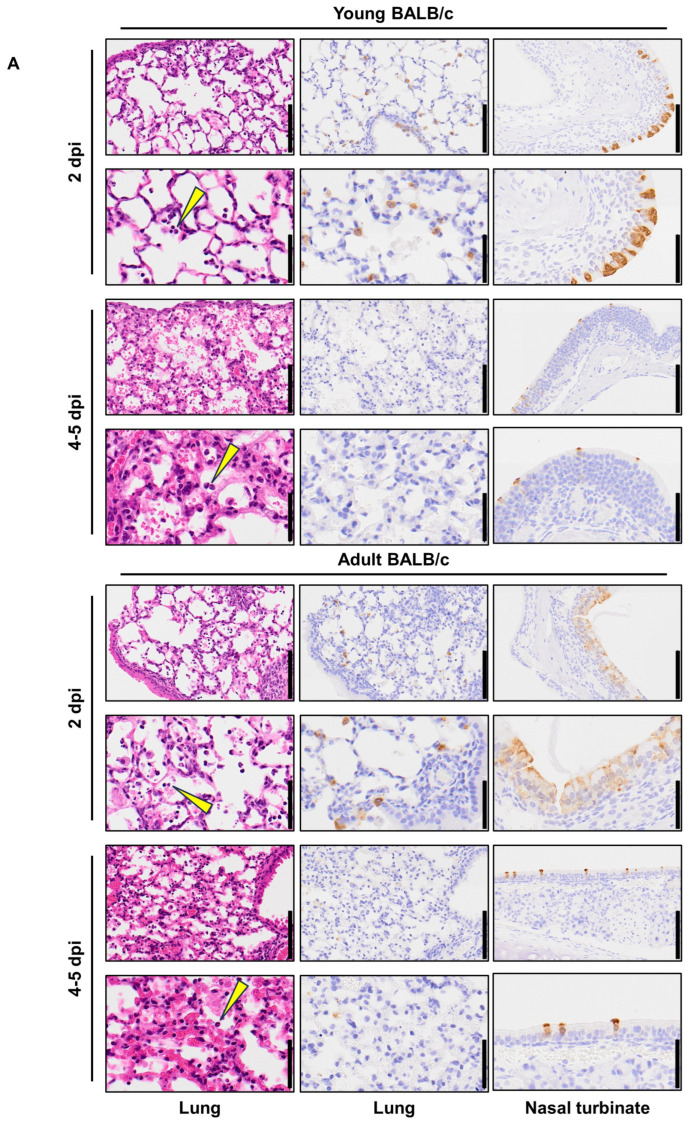

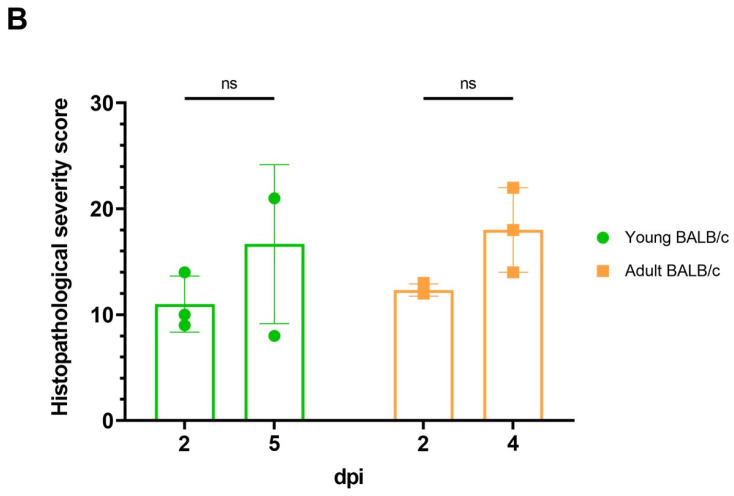
Pathological features of MASCV2-p9-infected mice. (**A**) Representative images of lungs (left and middle columns) and nasal turbinate (right columns) are shown. Left column, hematoxylin and eosin staining. Middle and right columns, immunohistochemistry using a rabbit polyclonal antibody that detects SARS-CoV-2 nucleocapsid protein. Low-magnification images (upper rows) and high-magnification images (lower rows) are shown. Arrow heads indicate inflammatory cells. Scale bars: 100 µm in low-magnification images; 50 µm in high-magnification images. (**B**) Histopathological scores of inflammation in the alveoli. Data shown are the mean scores ± 95% confidential interval. Each dot represents the score of each animal (n = 3). Data were analyzed using the Mann–Whitney test. ns, not significant.

To evaluate the replication and pathogenicity of MASCV2-p9 in BALB/c mice, we used two age groups of mice: 6-week-old (adolescent) and 18–20-week-old (mature adults). Mice were intranasally infected with 10^3^ PFU of MASCV2-p9. In the younger (6-week-old) and older (18-week-old) age groups, MASCV2-p9 exhibited high pathogenicity with MLD_50_ (mouse lethal dose 50; the dose required to kill 50% of infected mice) values of 10^3.3^ PFU and 10^2.7^ PFU, respectively (Figure 1B). The infection of mice with MASCV2-p9 resulted in high virus titers in the nasal turbinates and lungs in both the younger and older (20-week-old) age groups at Day 2 post-infection (Figure 1C). No substantial difference in viral titers in the respiratory organs at this timepoint was observed. The viruses were also recovered from the brain, heart, liver, and/or spleen (note: the brain samples collected for virus titration included the olfactory bulb). In the younger age group at Day 5 post-infection, MASCV2-p9 was detected in the respiratory organs of the infected animals at a titer of more than 1.02 × 10^5^ PFU/g, but the virus was not detected in any other organs tested. Given the greater reduction in body weight observed in infected adult mice compared to young mice, organ sampling was conducted on Day 4 post-infection rather than Day 5 post-infection. At Day 4 post-infection of the older mice, MASCV2-p9 was detected at a titer of more than 9.84 × 10^5^ PFU/g in the respiratory organs. Additionally, MASCV2-p9 was found in the brains of these animals at Day 4 post-infection. We then examined the histopathologic changes in the organs of the younger and older (20-week-old) age groups after MASCV2-p9 infection. A histopathological analysis revealed the infiltration of inflammatory cells such as neutrophils and mononuclear cells into the alveolar regions, accompanied with fibrinous exudates, in the lungs of both young and adult BALB/c mice at 2 days post-infection with MASCV2-p9 (Figure 2A). At the same timepoint, a viral antigen was detected by immunohistochemistry mainly on alveolar epithelial cells in both groups. At Day 5 (young) or Day 4 (adult) post-infection, there was a mild exacerbation of the histopathological inflammation score for both groups, although this alteration was not statistically significant (Figure 2B). Additionally, clear decreases in the number of virus-positive cells were observed in both groups. Virus-positive epithelial cells were also observed in the nasal turbinate of both groups at all timepoint examined with a clear decrease in the number of positive cells over time (Figure 2A). No virus-positive cells or significant morphological changes were detected histopathologically in the brain, heart, liver, spleen, kidney, or intestine.

### 3.2. Characterization of a Mouse-Adapted Strain of SARS-CoV-2 in C57BL/6J Mice

**Figure 3:**
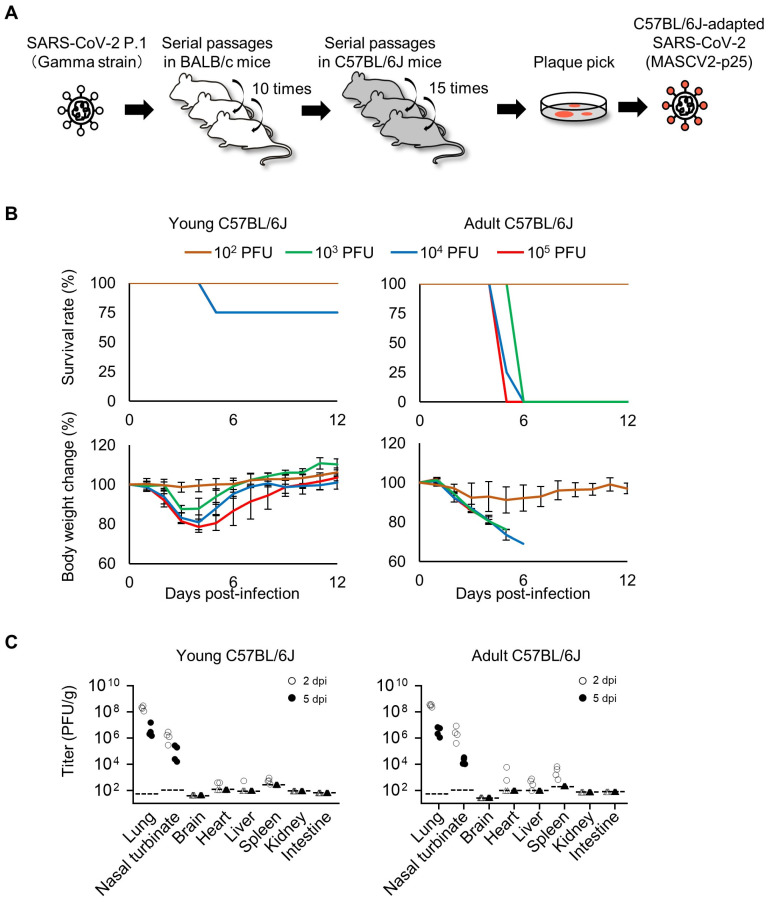
Virulence of MASCV2-p25 in C57BL/6J mice. (**A**) Schematic image of SARS-CoV-2 adaptation to C57BL/6J mice. (**B**) Survival rate and body weight changes. Four mice per group of young (6-week-old) or adult (24-week-old) C57BL/6J mice were infected with 10^1^ to 10^5^ PFU of MASCV2-p25, and survival and body weight were monitored daily for 12 days. The results are expressed as the mean ± SD. (**C**) Virus titers in organs of MASCV2-p25-infected mice. Four mice per group were euthanized and virus titers in the lungs, nasal turbinate, brain, heart, liver, spleen, kidney, and intestine were determined using plaque assays in VeroE6/TMPRSS2 cells. Dashed lines in the panels indicate the detection limit of the assay for each organ. Triangles in the panels indicate that the value was below the detection limit.

**Figure 4:**
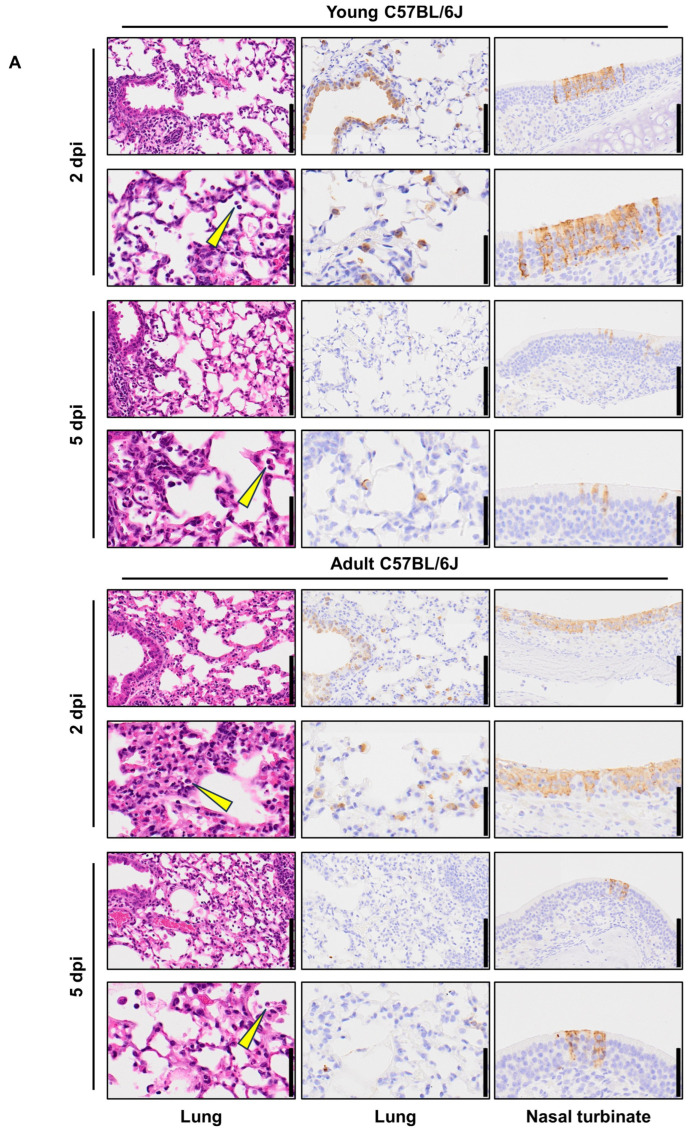

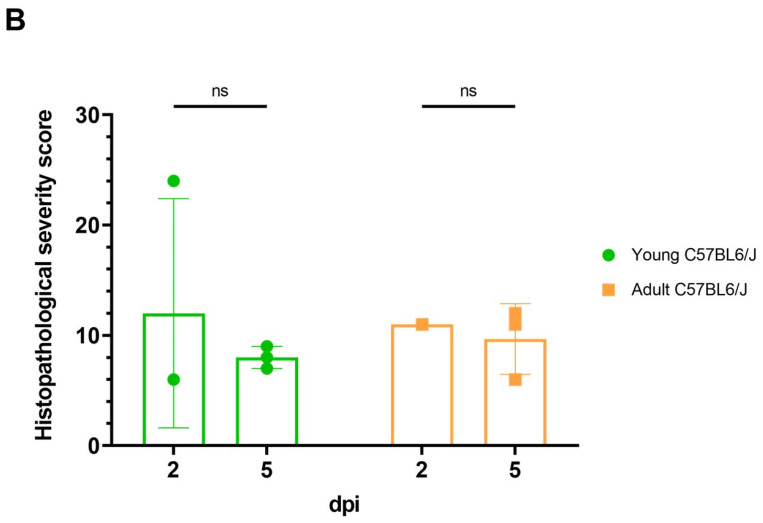
Pathological features of MASCV2-p25-infected mice. (**A**) Representative images of lungs (left and middle columns) and nasal turbinate (right columns) are shown. Left column, hematoxylin and eosin staining. Middle and right columns, immunohistochemistry using a rabbit polyclonal antibody that detects SARS-CoV-2 nucleocapsid protein. Low-magnification images (upper rows) and high-magnification images (lower rows) are shown. Arrow heads indicate inflammatory cells. Scale bars: 100 µm in the low-magnification images; 50 µm in high-magnification images. (**B**) Histopathological scores of inflammation in the alveoli. Data shown are the mean scores ± 95% confidential interval. Each dot represents the score of each animal (n = 2 or 3). Data were analyzed using the Mann–Whitney test. ns, not significant.

To acquire SARS-CoV-2 that causes severe pneumonia in BALB/c mice and C57BL/6J mice, the virus that was passaged in the lungs of BALB/c mice was passaged one more time (10 passages in total), and then passaged 15 times in the lungs of C57BL/6J mice (Figure 3A) (Table 1). After these serial passages, the virus in the lung homogenates was plaque-purified and amplified on VeroE6/TMPRSS2 cells. Sequencing analysis revealed that the C57BL/6J-adapted strain possesses a T295I substitution in NSP4, an F294L substitution in NSP5, an R39K in NSP9, a Q493K substitution in the S protein, and a T7I substitution in M (Table 2). The virus was designated MASCV2-p25. MASCV2-p25 showed high pathogenicity in adult (24-week-old) C57BL/6J mice and young (6-week-old) BALB/c mice, (MLD_50_: 10^2.5^ PFU for both), but low pathogenicity in young (6-week-old) C57BL/6J mice (MLD_50_: >10^5^ PFU) (Figure 3B and Supplementary Figure S1). MASCV2-p25 replicated well in the nasal turbinate and lungs of the young and adult C57BL/6J mice at 2 days post-infection (Figure 3C). Viruses were also recovered from the heart, liver, and spleen, but not brain, kidney, or intestines. On Day 5 post-infection, the virus was detected in the respiratory organs of infected young and adult C57BL/6J mice, but not in any other organs tested. No obvious difference in viral titers in the respiratory organs on Days 2 and 5 post-infection was found between the two age groups. The viral titers in the heart, liver, and spleen on Day 2 post-infection were generally higher in the infected older mice compared to the younger mice. A histopathological analysis demonstrated that alveolar inflammation, similar to that observed in BALB/c mice, was present in the lungs of both young and adult C57BL/6J mice on Day 2 post-infection with MASCV2-p25 (Figure 4A). At the same timepoint, the immunohistochemistry revealed that the viral protein was present mainly in the bronchiolar epithelial cells of both groups. The histopathological score of alveolar inflammation was comparable on Days 2 and 5 post-infection (Figure 4B), although fewer cells were positive for the viral protein on Day 5 than on Day 2 post-infection. Virus-positive epithelial cells were detected in the nasal turbinate of both groups at all timepoints examined, and fewer positive cells were observed on Day 5 post-infection compared with Day 2 post-infection (Figure 4A). No virus-positive cells or significant morphological changes were detected histopathologically in the brain, heart, liver, spleen, kidney, or intestines.

### 3.3. Characterization of a Mouse-Adapted Reporter SARS-CoV-2 in C57BL/6J Mice

**Figure 5:**
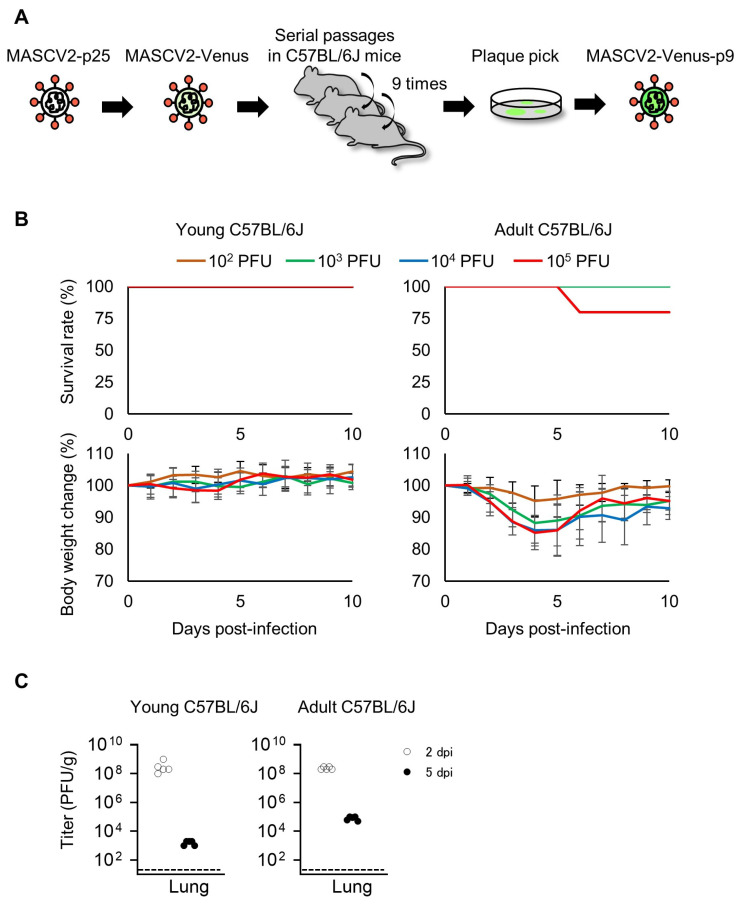
Virulence of MASCV2-Venus-p9 in C57BL/6J mice. (**A**) Schematic image of the generation of the mouse-adapted reporter SARS-CoV-2. (**B**) Survival rate and body weight changes. Five mice per group of young (6-week-old) or adult (24-week-old) C57BL/6J mice were infected with 10^2^ to 10^5^ PFU of MASCV2-Venus-p9, and survival and body weight were monitored daily for 10 days. The results are expressed as the mean ± SD. (**C**) Virus titers in the lungs of MASCV2-Venus-p9-infected mice. Five mice per group were euthanized and virus titers in the lungs were determined by using plaque assays in VeroE6/TMPRSS2 cells. Dashed lines in the panels indicate the detection limit of the assay.

**Figure 6:**
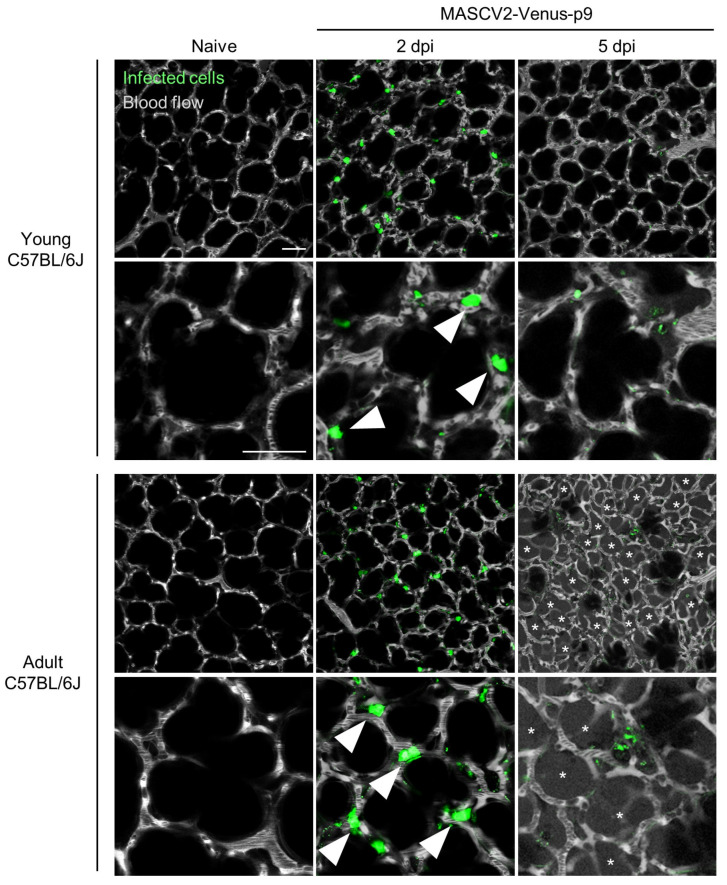
Two-photon in vivo imaging of SARS-CoV-2-infected C57BL/6J mice. Virus titers in organs of MASCV2-p25-infected mice. Mice were intranasally infected with MASCV2-Venus-p9 and observed on different days post-infection (dpi). Green indicates virus-infected cells. At the indicated timepoints, fluorescent dextran (white) was i.v. administered to visualize the lung architecture. Asterisks indicate areas where fluorescent dextran is leaking from the blood vessels into the alveoli. Low-magnification images (upper rows) and high-magnification images (lower rows) are shown. Scale bars, 50 µm. Arrow heads indicate type II alveolar epithelial cells.

To generate SARS-CoV-2, which causes severe pneumonia in C57BL/6J mice and expresses a fluorescent reporter protein in infected cells, the fluorescent reporter gene Venus and a porcine tescherovirus (PTV-1) 2A proteolytic cleavage site was inserted upstream of the N gene of MASCV2-p25, as described previously [33], using reverse genetics (named MASCV2-Venus) (Supplementary Figure S2) (Table 1). MASCV2-Venus exhibited reduced virulence in C57BL/6J mice compared with MASCV2-p25; therefore, to promote additional adaptation to the C57BL/6J mouse strain, we conducted serial lung-to-lung passages in C57BL/6J mice and generated a mouse-adapted reporter SARS-CoV-2 (named MASCV2-Venus-p9) (Figure 5A) (Table 1). The sequencing analysis revealed that the MASCV2-Venus-p9 strain possesses a V233I substitution in NSP4 and an L83F substitution in NSP13 (Table 2). In addition, MASCV2-Venus-p9 possessed the a28262g mutation in the non-coding region between ORF8 and the Venus gene. Although this mutation does not cause an amino acid substitution, this site serves as a translation regulatory sequence (TRS) that is important for gene transcription [37]. Within the TRS, there is a highly conserved core sequence (ACGAAC for SARS-CoV-2; nucleotide positions 28260–65 in TY7-501). When we inserted the Venus gene, as described by others [33], the TRS core sequence before the N gene in the MASCV2-Venus was ACAAAC, which is not the authentic TRS core sequence. However, during the passage of MASCV2-Venus in mice, the a-to-g transition occurred, resulting in the formation of the authentic TRS core sequence. In 24-week-old C57BL/6J mice, MASCV2-Venus-p9 caused significant weight loss without causing mortality. In contrast, young B6 mice infected with MASCV2-Venus-p9 showed no weight loss (Figure 5B). The infection of C57BL/6 mice with MASCV2-Venus-p9 resulted in high virus titers in the lungs in both the younger and older (24-week-old) age groups at Day 2 post-infection (Figure 5C). The viral load of MASCV2-Venus-p9 at Day 2 of infection was comparable to that of MASCV2-p25 in both young and adult mice (Figure 3C and Figure 5C). In contrast, the viral load of MASCV2-Venus-p9 at Day 5 of infection tended to be lower than that of MASCV2-p25 in both the young and adult groups. To observe the pathological changes in SARS-CoV-2-infected lungs in living animals, we examined MASCV2-Venus-p9-infected C57BL/6J mice using an in vivo imaging system that we established previously [11,34]. Two days after infection, large numbers of infected cells expressing Venus were found in the lungs of both young and adult B6 mice. At 5 days post-infection, a granular Venus signal was observed, indicating that the infected cells had disintegrated (Figure 6). In contrast, fluorescence-labelled dextran leaked from the blood vessels into the alveoli in the lungs of adult C57BL/6J mice infected with MASCV2-Venus-p9 on Day 5 of infection, indicating that increased vascular permeability associated with tissue damage had occurred.

## 4. Discussion

We observed that MASCV2-p25 replicated efficiently in the lungs of C57BL/6J mice, causing fatal pneumonia. The histopathologic analysis revealed the severe inflammation and infiltration of immune cells in the lungs of C57BL/6J mice infected with MASCV2-p25, similar to that commonly reported for COVID-19 patients with severe pneumonia. In addition, we established a mouse-adapted reporter SARS-CoV-2 that causes severe lung inflammation in adult C57BL/6J mice. Furthermore, MASCV2-Venus-p9 fluorescently labeled infected cells in the lungs of C57BL/6J mice with sufficient fluorescence for in vivo two-photon imaging. The C57BL/6 mouse is the most widely used background strain for the development of mouse models of obesity, diabetes, hypertension, and immune disorders, which are conditions associated with an increased risk of severe COVID-19 [1,2]. Therefore, MASCV2-Venus-p9 represents a useful tool for studies of the pathogenesis of severe COVID-19 and the development of therapeutic agents for severe pneumonia.

The parental strain, TY7-501, has the N501Y spike substitution, which is associated with mouse adaptation [30,38]. MASCV2-p9 and MASCV2-p25 contain the Q493R and Q493K substitutions in the RBD (receptor-binding domain), respectively, in addition to the N501Y substitution. Previous studies have reported that a mutation at position 493 in the RBD may enhance the binding affinity of the S protein for the murine ACE2 receptor [25,36,39]. Together, these findings suggest that the Q493R or Q493K substitution in the RBD of MA strains along with the N501Y substitution may increase the binding affinity of the S protein for murine ACE2, thereby allowing a virus carrying Q493R/N501Y or Q493K/N501Y in its Spike protein to replicate efficiently in the lungs of mice. Although an g-to-a transition was introduced in the TRS when MASCV2-Venus was generated by inserting the Venus gene into MASCV2-p25, this base change in the TRS reverted back in MASCV2-Venus-p9 after further adaptation to C57BL/6J. Therefore, it is possible that the reversion of this mutation in the TRS is responsible for the increased proliferation of MASCV2-Venus-p9. Compared to MASCV2-p25, MASCV2-Venus-p9 contains the substitutions V233I in NSP4 and L83F in NSP13, which are thought to increase the proliferative potential of the virus in C57BL/6J mice. NSP4, which has a transmembrane domain, and NSP13, which has helicase activity, are viral proteins involved in viral replication [40], and it is possible that substitution-induced functional changes in these proteins directly enhance viral replication. Further studies are needed to determine how substitutions in these viral proteins contribute to the proliferative potential of MASCV2-Venus-p9.

In vivo imaging using two-photon microscopy and MASCV2-Venus-p9 allows us to observe cell behavior, morphological changes, and blood flow dynamics in living animals infected with SARS-CoV-2. In contrast, a conventional histological analysis provides static images at a higher resolution. The use of both of these histological approaches to analyze samples from the same animal would provide more detailed insights into the pathogenesis of SARS-CoV-2.

In conclusion, MASCV2-Venus-p9 is a powerful and versatile tool for studying SARS-CoV-2 pathogenicity in vivo.

## Figures and Tables

**Table 1:** Summary of the processes used to produce the virus strains in this study.

Viral Strain	Production Process
MASCV2-p9	MASCV2-p9 was derived from TY7-501 by sequential lung-to-lung passages in BALB/c mice (9 passages).
MASCV2-p25	MASCV2-p25 was derived from TY7-501 by sequential lung-to-lung passages in BALB/c mice (10 passages) followed by sequential lung-to-lung passages in C57BL/6J mice (15 passages).
MASCV2-Venus	MASCV2-Venus was generated by inserting the fluorescent gene Venus into the genome of MASCV2-p25 by use of reverse genetics.
MASCV2-Venus-p9	MASCV2-Venus-p9 was derived from MASCV2-Venus by sequential lung-to-lung passages in C57BL/6J mice (9 passages).

**Table 2:** Amino acid substitutions and nucleotide changes detected in MASCV2-p9, MASCV2-p25, MASCV2-Venus, and MASCV2-Venus-p9 compared with TY7-501. - indicates no mutation in the corresponding genome sequence or amino acid compared to TY7-501.

MASCV2-p9	Amino acid substitution	-	-	-	NSP4 A307V	NSP5 F294L	-	-	S Q493R	-	-	-	ORF7a T14I	-
	Gene mutation	-	-	-	c9474t	t10934c	-	-	a23040g	-	-	-	c27434t	-
MASCV2-p25	Amino acid substitution	-	-	NSP4 T295I	-	NSP5 F294L	NSP9 R39K	-	S Q493K	-	-	M T7I	-	-
	Gene mutation	t2365c	-	c9438t	-	t10934c	g12801a	-	c23039a	t24706c	-	c26542t	-	-
MASCV2-Venus	Amino acid substitution	-	-	NSP4 T295I	-	NSP5 F294L	NSP9 R39K	-	S Q493K	-	-	M T7I	-	-
	Gene mutation	-	-	c9438t	-	t10934c	g12801a	-	c23039a	-	-	c26542t	-	g28262a
MASCV2-Venus-p9	Amino acid substitution	-	NSP4 V233l	NSP4 T295I	-	NSP5 F294L	NSP9 R39K	NSP13 L83F	S Q493K	-	-	M T7I	-	-
	Gene mutation	-	g9251a	c9438t	-	t10934c	g12801a	g16476t	c23039a	-	c24904t	c26542t	-	-

## Data Availability

Data are contained within the article.

## Supplementary Materials

The following supporting information can be downloaded at: https://www.mdpi.com/article/10.3390/v16040537/s1, Figure S1: Pathogenicity of MASCV2-p25 in young BALB/c mice.; Figure S2: Schematic representation of the BAC used to generate MASCV2-Venus.
