# Supplementary material for: Development of a Mouse-Adapted Reporter SARS-CoV-2 as a Tool for Two-Photon In Vivo Imaging
Source: Viruses. 2024 Mar 29;16(4):537. doi: 10.3390/v16040537 (PMC11053786; doi:10.3390/v16040537)
Supplement: Supplementary file 1 [file viruses-16-00537-s001.zip › Supplementary Figure S1.pdf]

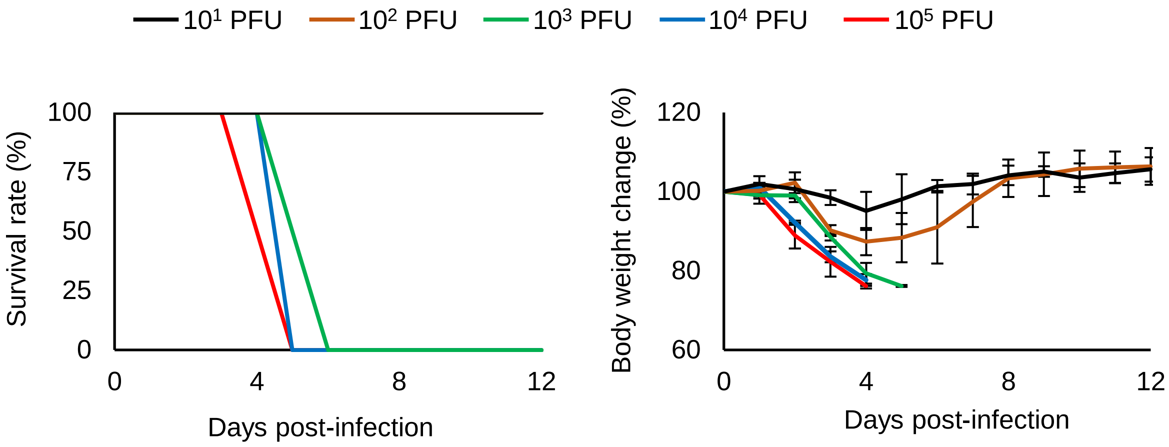

Supplementary Figure S1. Pathogenicity of MASCV2-p25 in young BALB/c mice. Survival rate (left panel) and body weight changes (right panel) of MASCV2-p25-infected mice. Four mice per group of young (6-week-old) BALB/c mice were intranasally infected with 10^1 to 10^5 PFU of MASCV2-p25, and survival and body weight were monitored daily for 12 days. The results are expressed as the mean ± SD.
