# Supplementary figures and images for: Development of a Mouse-Adapted Reporter SARS-CoV-2 as a Tool for Two-Photon In Vivo Imaging
Source: Viruses. 2024 Mar 29;16(4):537. doi: 10.3390/v16040537 (PMC11053786; doi:10.3390/v16040537)

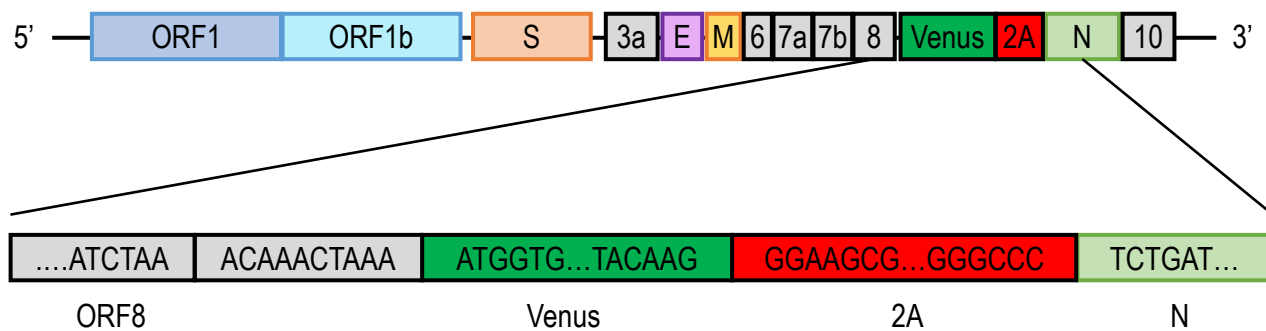

**Supplementary Figure S2.** Schematic representation of the BAC used to generate MASCV2-Venus.

Supplement: Supplementary file 1 [file viruses-16-00537-s001.zip › Supplementary Figure S2.pdf]
